# A Low-g MEMS Accelerometer with High Sensitivity, Low Nonlinearity and Large Dynamic Range Based on Mode-Localization of 3-DoF Weakly Coupled Resonators

**DOI:** 10.3390/mi12030310

**Published:** 2021-03-16

**Authors:** Muhammad Mubasher Saleem, Shayaan Saghir, Syed Ali Raza Bukhari, Amir Hamza, Rana Iqtidar Shakoor, Shafaat Ahmed Bazaz

**Affiliations:** 1 Department of Mechatronics Engineering, National University of Sciences and Technology, Islamabad 44000, Pakistan; shayaan.saghir18@mts.ceme.edu.pk (S.S.); saraza.mts19ceme@mts.ceme.edu.pk (S.A.R.B.); a.hamza@ceme.nust.edu.pk (A.H.); 2 National Centre of Robotics and Automation (NCRA), Islamabad 44000, Pakistan; rana.iqtidar@mail.au.edu.pk (R.I.S.); bazaz@case.edu.pk (S.A.B.); 3 Department of Mechatronics Engineering, Air University, Islamabad 44000, Pakistan; 4 Department of Electrical and Computer Engineering, Sir Syed CASE Institute of Technology, Islamabad 4400, Pakistan

**Keywords:** dynamic range, finite-element-method, MEMS accelerometer, mode-localization, mode-aliasing, nonlinearity, resolution, sensitivity tuning, SOIMUMPs, weakly coupled resonators

## Abstract

This paper presents a new design of microelectromechanical systems (MEMS) based low-g accelerometer utilizing mode-localization effect in the three degree-of-freedom (3-DoF) weakly coupled MEMS resonators. Two sets of the 3-DoF mechanically coupled resonators are used on either side of the single proof mass and difference in the amplitude ratio of two resonator sets is considered as an output metric for the input acceleration measurement. The proof mass is electrostatically coupled to the perturbation resonators and for the sensitivity and input dynamic range tuning of MEMS accelerometer, electrostatic electrodes are used with each resonator in two sets of 3-DoF coupled resonators. The MEMS accelerometer is designed considering the foundry process constraints of silicon-on-insulator multi-user MEMS processes (SOIMUMPs). The performance of the MEMS accelerometer is analyzed through finite-element-method (FEM) based simulations. The sensitivity of the MEMS accelerometer in terms of amplitude ratio difference is obtained as 10.61/g for an input acceleration range of ±2 g with thermomechanical noise based resolution of 0.22 $\mu g/\sqrt{\mathrm{Hz}}$ and nonlinearity less than 0.5%.

## 1. Introduction

Microelectromechanical systems (MEMS) accelerometers have been widely used in many different applications for the last three decades due to their small size, light weight, batch fabrication and low power requirements. The resonant MEMS accelerometers are considered to be ideal for precision navigation and seismic sensing applications due to their high sensitivity and resolution [1,2]. Most of the resonant MEMS accelerometers use resonant frequency shift, based on stiffness modulation, as an output metric for the measurement of input acceleration due to output signal being quasi-digital and high sensitivity [3,4]. However, the resonant frequency shift output metric for MEMS accelerometers is also strongly affected by the environmental variations including temperature and pressure [5,6]. This requires additional error compensation techniques for the stable operation of such resonant MEMS accelerometers [7,8].

Recently, a relatively new approach based on multiple coupled resonators and using resonator amplitude as output metric has been implemented for different MEMS resonators based sensing applications including force sensing [9], mass sensing [10], stiffness sensing [11] and displacement sensing [12]. These resonant MEMS sensors operate based on the mode-localization effect between the weakly coupled resonators. The mode-localization involves the confinement of the energy into any one of the MEMS resonators due to an input stiffness perturbation in the coupled resonator system. The corresponding changes in the amplitudes of the resonators are then used as an output metric for the physical parameter to be measured. The sensitivity of such resonant MEMS sensors is 2–3 orders of magnitude higher than the conventional resonant frequency shift based sensors [13].

One of the earliest work on the mode-localization based resonant MEMS accelerometers was presented by Zhang et al. [14]. Two double-ended-tuning-fork (DETF) resonators, weakly coupled through mechanical beams formed a 2-DoF system for the acceleration sensing. The amplitude ratio (AR) of the two resonators, in out-of-phase mode, was used as an output metric and results showed that the sensitivity of mode-localization based MEMS accelerometer, for an input range of ±1 g, is 1.26/g which is 302 times higher than that of conventional frequency shift based output metric. Yang et al. [15] modified the accelerometer design presented in [14] by using two single-tine weakly coupled resonators instead of DETF resonators to decrease the number of vibration modes and implement closed-loop readout sensing. This allowed to achieve the high resolution and amplitude ratio sensitivity of 1.32/g. To further increase the sensitivity, Kang et al. [16] used 3-DoF weakly coupled resonators using mechanical coupling beams and demonstrated an amplitude ratio sensitivity of 4.38/g which was 348% higher than the 2-DoF coupled resonator system presented earlier in [14]. Recently, Peng et al. [17] implemented the 4-DoF resonator system with 3-DoF weakly coupled resonators, using mechanical beams, attached in series and one electrically coupled resonator in parallel. The maximum amplitude ratio sensitivity is reported to be 23.37/g.

In the previous works presented on the mode-localization based resonant MEMS accelerometers, the amplitude ratio of the resonators is used as an output metric for acceleration measurement to achieve high sensitivity. However, the amplitude ratio nonlinearity is a strong function of the stiffness perturbation in the resonators for an input acceleration. The nonlinearity is maximum for low values of the stiffness perturbation around the veering zone and thus limits the measurement range of the MEMS accelerometer [14]. Recently, Pandit et al. [18] have presented a mode-localized MEMS accelerometer, for input acceleration from 0–1 g, using amplitude ratio difference (ARD) of 2-DoF weakly coupled single beam resonators that are attached on the two sides of the proof mass. The amplitude ratio difference sensitivity of 11/g is reported with enhanced linearity for low stiffness perturbation values.

In this paper, a new design of mode-localization based MEMS accelerometer is presented with two 3-DoF weakly coupled resonator systems attached on each side of proof mass for high sensitivity and large input range. The amplitude ratio difference of the two resonator system is implemented as an output metric for the input acceleration to minimize the nonlinearity for low input acceleration values. The proposed design utilizes electrostatic tuning of the 3-DoF resonators to increase the sensitivity.

## 2. Structural Design and Working Principle

**Figure 1:**
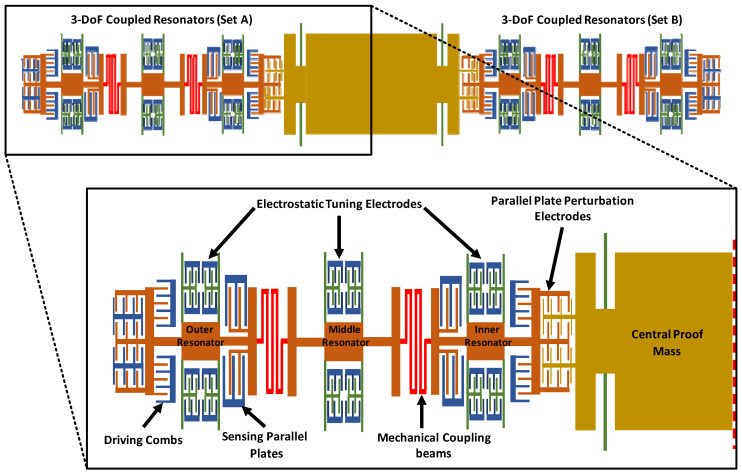
The structural design of mode-localized MEMS accelerometer with two sets of 3-DoF weakly coupled resonators on either side of central proof mass.

Figure 1 shows the schematic diagram of the MEMS accelerometer design with two sets of three weakly coupled resonators attached on either side of the central proof mass. Each of the resonator consists of four fixed-guided beams to minimize any out-of-plane bending and a relatively large central plate as compared to traditional MEMS resonators to avoid any variation in the mass value of resonators due to fabrication process tolerances. The outer and inner resonator of the 3-DoF resonator set have equal stiffness and mass and are set to vibration by using comb drive based electrostatic actuators. For the measurement of the vibration amplitude of these two resonators, capacitive sensing parallel plates are included in the design in differential current sensing configuration to minimize common mode signals. The mass of the middle resonator is same as the adjacent resonators but stiffness value of its suspension beams is higher. For the stiffness tuning of coupled resonators, electrostatic parallel plates based tuning electrodes are attached to each resonator. The three resonators are weakly coupled by using serpentine shaped mechanical suspension beams.

The central proof mass is free to move and is suspended by using four fixed-guided beams. The proof mass and inner most resonator, on each side of the proof mass, are mechanically separated but electrostatically coupled through parallel plate electrodes. For an input acceleration, the displacement in the central proof mass changes the electrostatic coupling gap between the proof mass and inner most resonator of 3-DoF resonator system on each side. This gap change leads to an electrical spring stiffness variation of the inner resonators and hence results in the localization of the energy and changes in the vibration amplitudes of the resonators. For the MEMS accelerometer, the difference in the amplitude ratios of the outer and inner resonator on each side of the central proof mass is used as an output metric for input acceleration measurement. The proposed MEMS accelerometer is designed by following the fabrication limitations of commercially available SOIMUMPs process with structural layer of Silicon and thickness of 25 µm [19]. Table 1 summarizes main design parameters of the MEMS accelerometer design.

## 3. Mathematical Model

**Figure 2:**
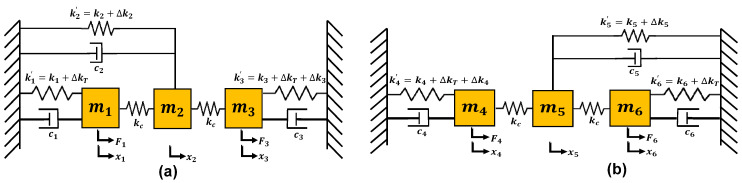
Lumped mass-spring-damper model of the 3-DoF weakly coupled MEMS resonators (**a**) Set A (**b**) Set B.

The working of the MEMS accelerometer can be described by using a lumped mass-spring-damper model for each 3-DoF resonators set attached on either side of the central proof mass, as shown in Figure 2. For the dual resonator drive of each resonator set, the equations of motion can be written as;

(1) $$m_{1}{\ddot{x}}_{1}+c_{1}{\dot{x}}_{1}+k_{1}^{\prime }x_{1}+k_{c}\left(x_{1}-x_{2}\right)=F_{1}cos\left(\omega t\right)$$

(2) $$m_{2}{\ddot{x}}_{2}+k_{2}^{\prime }x_{2}+k_{c}\left(x_{2}-x_{1}\right)+k_{c}\left(x_{2}-x_{3}\right)=0$$

(3) $$m_{3}{\ddot{x}}_{3}+c_{3}{\dot{x}}_{3}+k_{3}^{\prime }x_{3}+k_{c}\left(x_{3}-x_{2}\right)=F_{3}cos\left(\omega t\right)$$

(4) $$m_{4}{\ddot{x}}_{4}+c_{4}{\dot{x}}_{4}+k_{4}^{\prime }x_{4}+k_{c}\left(x_{4}-x_{5}\right)=F_{4}cos\left(\omega t\right)$$

(5) $$m_{5}{\ddot{x}}_{5}+k_{5}^{\prime }x_{5}+k_{c}\left(x_{5}-x_{4}\right)+k_{c}\left(x_{5}-x_{6}\right)=0$$

(6) $$m_{6}{\ddot{x}}_{6}+c_{6}{\dot{x}}_{6}+k_{6}^{\prime }x_{6}+k_{c}\left(x_{6}-x_{5}\right)=F_{6}cos\left(\omega t\right)$$

where the $m_{i}$ (for *i* = 1 to 6) is the masses of the resonators, $c_{i}$ (for *i* = 1 to 6) is the damping coefficient due to slide and squeeze film air damping, $x_{i}$ (for *i* = 1 to 6) is the displacement in the resonators, $F_{1}$ and $F_{3}$ are the driving forces acting on the outer and inner resonator in the resonator set A, $F_{4}$ and $F_{6}$ are the driving forces acting on the inner and outer resonator in the resonator set B and ω is the frequency of oscillation. The $k_{c}$ is the stiffness of weakly coupled mechanical springs and stiffness values$\;k_{1}^{\prime }$, $k_{2}^{\prime }$, $k_{3}^{\prime }$, $k_{4}^{\prime }$, $k_{5}^{\prime }$ and $k_{6}^{\prime }$ can be written as;

(7) $$k_{1}^{\prime }=k_{1}+∆k_{T}$$

(8) $$k_{2}^{\prime }=k_{2}+∆k_{T2}$$

(9) $$k_{3}^{\prime }=k_{3}+∆k_{T}+∆k_{3}$$

(10) $$k_{4}^{\prime }=k_{4}+∆k_{T}+∆k_{4}$$

(11) $$k_{5}^{\prime }=k_{5}+∆k_{T2}$$

(12) $$k_{6}^{\prime }=k_{6}+∆k_{T}$$

where $k_{i}$ (*i* = 1 to 6) are the mechanical stiffness values of the suspension beams attached to the six resonators, $∆k_{T}$ and $∆k_{T2}$ are stiffness changes corresponding to the tuning voltage applied to the inner-outer resonators and middle resonator respectively. The $∆k_{3}$ and $∆k_{4}$ are stiffness perturbations in the inner resonators in the resonator set A and B respectively. These stiffness perturbations are induced in the left and right side 3-DoF resonator systems corresponding to proof mass displacement for an input acceleration. Since the two sets of 3-DoF resonator systems on each side of the proof mass are identical, the general solution for the resonator set A is discussed and can be written in the matrix form as;

(13) $$\left[M\right]\ddot{X}+\left[C\right]\dot{X}+\left[K\right]X=\left[F\right]$$

(14) $$\left[\begin{array}{ccc} m_{1} & 0 & 0\\ 0 & m_{2} & 0\\ 0 & 0 & m_{3} \end{array}\right]\left[\begin{array}{c} {\ddot{x}}_{1}\\ {\ddot{x}}_{2}\\ {\ddot{x}}_{3} \end{array}\right]+\left[\begin{array}{ccc} c_{1} & 0 & 0\\ 0 & c_{2} & 0\\ 0 & 0 & c_{3} \end{array}\right]\left[\begin{array}{c} {\dot{x}}_{1}\\ {\dot{x}}_{2}\\ {\dot{x}}_{3} \end{array}\right]+\left[\begin{array}{ccc} k_{1}^{\prime }+k_{c} & -k_{c} & 0\\ -k_{c} & k_{2}^{\prime }+2k_{c} & -k_{c}\\ 0 & -k_{c} & k_{3}^{\prime }+k_{c} \end{array}\right]\left[\begin{array}{c} x_{1}\\ x_{2}\\ x_{3} \end{array}\right]=\left[\begin{array}{c} F_{1}cos\left(\omega t\right)\\ 0\\ F_{3}cos\left(\omega t\right) \end{array}\right]$$

assuming that the solution of the system is of following form;

(15) $$\left[X\right]=\left[A\right]\sin\left(\omega t\right)+\left[B\right]\cos\left(\omega t\right)$$

where the vectors $\left[A\right]=\left[\begin{array}{c} a_{1}\\ a_{2}\\ a_{3} \end{array}\right]$ and $\left[B\right]=\left[\begin{array}{c} b_{1}\\ b_{2}\\ b_{3} \end{array}\right]$. By using the above solution in Equation (14) yields the following results;

(16) $$\left[T\right]\left[\begin{array}{c} \left[A\right]\\ \left[B\right] \end{array}\right]=\left[\begin{array}{c} \left[F\right]\\ \left[0\right] \end{array}\right]$$

where *T* is given by

(17) $$\left[T\right]=\left[\begin{array}{cc} \left[K\right]-\omega^{2}\left[M\right] & -\omega \left[C\right]\\ \omega \left[C\right] & \left[K\right]-\omega^{2}\left[M\right] \end{array}\right]$$

After *A* and *B* are found by using Equation (16), the frequency of the ith resonator (i = 1 to 6) can be obtained as:

(18) $$x_{i}\left(\omega \right)=\sqrt{A_{i}+B_{i}}$$

(19) $$tan\theta_{i}=-\frac{B_{i}}{A_{i}}$$

To sense the input acceleration, the amplitude ratios of rL=x1x3 (for the resonator Set A) and rR=x4x6 (for the resonator Set B) are utilized and the output metric is the difference of these two ratios i.e.,

(20) $$\mathrm{output}\;=|r_{L}-r_{R}|$$

For the operation of the 3-DoF system, first two modes are generally preferred. In the first mode, the two outer resonators vibrate in-phase with each other while they vibrate out-of-phase in the second mode. Assuming that no damping is present in the system and $k_{1}=k_{3}=k$, $m_{1}=m_{3}=m$ and $∆k\ll k_{3}^{\prime }$, the natural frequencies of these two modes can be expressed as:

(21) $$\omega_{ip}=\sqrt{\frac{1}{m}\left[\left(k+∆k_{T}\right)+k_{c}+\frac{1}{2}\left(∆k_{3}-2\frac{2k}{\gamma }-\sqrt{∆k_{3}{}^{2}+{\left(\frac{2k}{\gamma }\right)}^{2}}\right)\right]}$$

(22) $$\omega_{op}=\sqrt{\frac{1}{m}\left[\left(k+∆k_{T}\right)+k_{c}+\frac{1}{2}\left(∆k_{3}-2\frac{2k}{\gamma }+\sqrt{∆k_{3}{}^{2}+{\left(\frac{2k}{\gamma }\right)}^{2}}\right)\right]}$$

where $\omega_{ip}$ and $\omega_{op}$ represent the resonant frequency values of the in-phase and out-of-phase modes, respectively. The term γ in above equations is given by;

(23) $$\gamma =\frac{\left(k+∆k_{T}\right)\left[k_{2}^{\prime }-\left(k+∆k_{T}\right)+k_{c}\right]}{k_{c}^{2}}$$

For the design of 3-DoF weakly coupled resonators system, the following criteria is fulfilled [20];

(24) $$k_{2}^{\prime }>2k_{1}^{\prime }$$

(25) $$k_{c}<\frac{k_{1}^{\prime }}{10}<\frac{k_{2}^{\prime }}{20}$$

## 4. Performance Analysis of the MEMS Accelerometer Design

### 4.1. Natural Frequency Analysis

**Figure 3:**
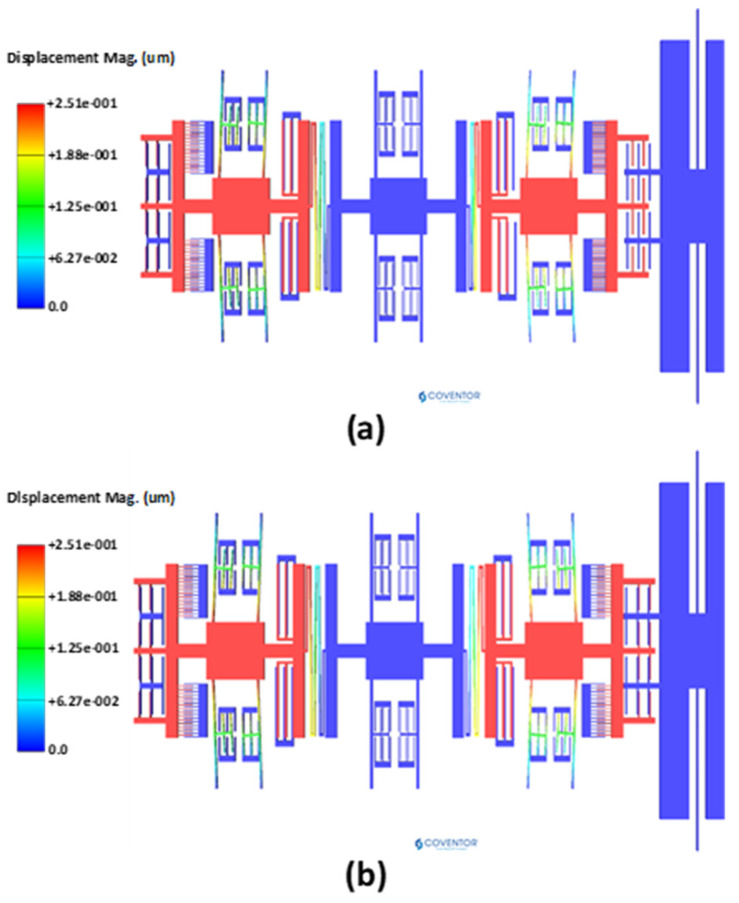
Natural frequency analysis for the MEMS accelerometer (**a**) in-phase mode (12,220 Hz) (**b**) out-of-phase mode (12,223.3 Hz).

The natural frequency analysis and corresponding mode shapes of the MEMS accelerometer are analyzed by behavioral model based FEM simulations in the CoventorWare software. Figure 3a shows the in-phase and out-of-phase mode shapes of the 3-DoF MEMS resonators, in Set A of the MEMS accelerometer, with the natural frequency values of 12,220.048 Hz and 12,223.264 Hz respectively. In the in-phase mode, both the inner and outer resonator vibrate in the same direction while in the out-of-phase mode both resonators vibrate in the opposite direction.

### 4.2. Stiffness Perturbation in the the Inner Resonators of 3-DoF Resonator Sets

The proof mass of the MEMS accelerometer is electrostatically coupled to the inner resonators in the resonator Set A and Set B. The change in the stiffness of these inner resonators corresponding to the input acceleration g can be expressed as;

(26) $$∆k_{3}=-\frac{N\varepsilon A}{{\left(d+\frac{m_{p}a}{k_{p}}-\frac{m_{3}a}{k_{3}}+x_{g}\right)}^{3}}{\left(∆V\right)}^{2}$$

(27) $$∆k_{4}=-\frac{N\varepsilon A}{{\left(d-\frac{m_{p}a}{k_{p}}+\frac{m_{4}a}{k_{4}}-x_{g}\right)}^{3}}{\left(∆V\right)}^{2}$$

where *N* is the number of parallel plate capacitive perturbation electrodes, ε is the permittivity, *A* is the overlap area of electrodes, *d* is the initial gap between the electrodes, $m_{p}$ is the mass of the proof mass, $k_{p}$ is the stiffness of the suspension beams attached to the proof mass, $m_{3}$ and $m_{4}$ are the mass values for inner resonators in the resonator Set A and B respectively, $k_{3}$ and $k_{4}$ is the stiffness of the inner resonators in the resonator Set A and B respectively, a is the input acceleration and ∆V is the potential difference between the resonators and proof mass. The term $x_{g}$ is due to the DC voltage based displacement applied to the resonators and is given as:

(28) $$x_{g}=\frac{0.5\varepsilon_{0}A{\left(∆V\right)}^{2}}{k_{p}}\;\left(\frac{1}{{\left(d-\frac{m_{p}a}{k_{p}}+\frac{m_{3}a}{k_{3}}\right)}^{2}}-\frac{1}{{\left(d+\frac{m_{p}a}{k_{p}}-\frac{m_{4}a}{k_{4}}\right)}^{2}}\right)$$

**Figure 4:**
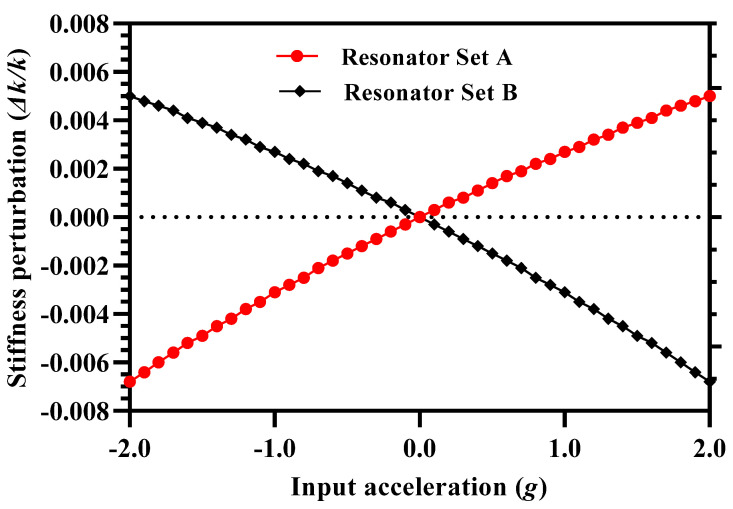
Normalized stiffness perturbation in the inner resonators of 3-DoF resonator sets for an input acceleration in the range of ±2 g.

The effect of input acceleration in the range of ±2 g on the normalized stiffness change in the inner coupling resonators is studied through FEM simulations. The inner and outer resonators in the two sets of 3-DoF resonators are oscillated in the out-of-phase mode at 35 V DC and 15 mV AC voltage. In the simulations, the proof mass and stator plates of perturbation electrodes are kept at ground potential. The value of the air damping is computed using DampingMM module in Coventorware for an air pressure of 10 mTorr and room temperature [21]. At zero input acceleration the effective spring constant of the inner and outer resonators is equal with a value of 40 N/m. For an applied acceleration, the stiffness of inner resonators, attached on each side of the mass, changes. Figure 4 shows that the effect of input acceleration in the range of ±2 g on the normalized stiffness perturbation in the inner resonators (∆k/k) is nearly linear. However, the results shows that the ∆k/k values for inner resonators in both the resonator Set A and B differ with a maximum value of 0.0018 at input acceleration of ±2 g. This is due to the fact that for a given acceleration, if the gap in the perturbation electrodes increases in inner resonator in Set A, it equally decreases in the inner resonator in Set B and from Equations (26) and (27) it is clear that it will result in a small difference in the ∆k/k for both the inner resonators.

### 4.3. Dynamic Response and Mode Localization

**Figure 5:**
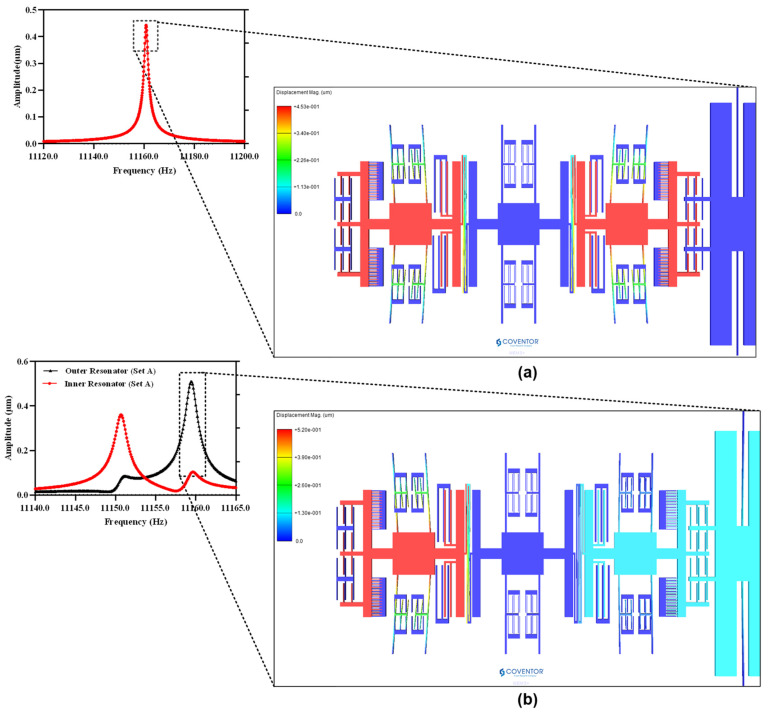
Frequency response of the 3-DoF weakly coupled resonators in Set A (**a**) at 0 g with AR = 1 (**b**) at 0.5 g with AR = 6.2.

For the mode-localization analysis, both the outer and inner resonators of 3-DoF coupled resonator system are set into vibration in the out-of-phase mode. Figure 5a shows the frequency response of Set A 3-DoF resonator system at out-of-phase mode resonance frequency and with input acceleration of 0 g (∆k = $∆k_{3}=0)$. The results show that at resonant frequency of 11,160.83 Hz, both the outer and inner resonator vibrate with the same amplitude of 0.44 µm and have an amplitude ratio of 1. Moreover, since the stiffness of both the inner and outer resonators is equal and there is no stiffness perturbation, only the out-of-phase mode is activated and in-phase mode is fully restrained. Figure 5b shows the frequency response of the same system at −0.5 g ((∆k = $∆k_{3}$ = −0.0587). The results show that at −0.5 g, the negative stiffness perturbation introduced by the proof mass due to electrostatic coupling results in mode-localization. The in-phase and out-of-phase resonant frequencies are separated by 8.91 Hz with frequency values of 11,150.53 Hz and 11,159.43 Hz respectively. At in-phase resonant frequency, the outer and inner resonator vibrate with the amplitude of 0.1013 µm and 0.5138 µm respectively and the amplitude ratio value is 0.197. At out-of-phase resonant frequency, the amplitude ratio of outer and inner resonator in Set A is 4.955 with corresponding vibration amplitudes of 0.510 µm and 0.103 µm respectively.

### 4.4. Mode Aliasing and Frequency Based Sensitivity Analysis

**Figure 6:**
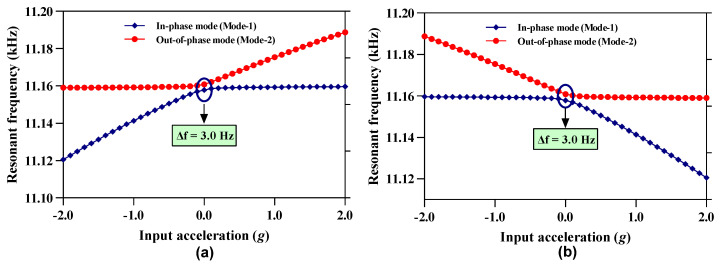
In-phase and out-of-phase mode resonant frequency variation with input acceleration (**a**) Resonator Set A (**b**) Resonator Set B.

The bandwidth of resonant modes in the coupled 3-DoF resonator systems is strongly dependent on the quality factor and at low air pressure both the in-phase and out-of-phase modes may overlap and hence result in mode aliasing. For the 3-DoF weakly coupled resonator system, the frequency difference $\left(∆f\right)$ between the in-phase and out-of-phase mode must be greater than $2∆f_{3dB}$ to avoid mode-aliasing [22]. Figure 6a,b shows the frequency variation in both the in-phase and out-of-phase mode frequency for the resonator Set A and Set B respectively for an input acceleration in the range of ± 2 g. The results show that in the resonator Set A, both the in-phase and out-of-phase resonant frequency values are separated by a minimum frequency of 3.0 Hz at 0 g input acceleration i.e., at ∆k/k = 0. The frequency difference between the two resonant modes increases as the input acceleration changes from 0 to ±2 g. The behavior of frequency difference variation with respect to input acceleration is similar in 3-DoF resonator Set B. For the MEMS accelerometer, the $2∆f_{3dB}$ value at 0 g is 2.92 Hz which is less than the ∆f = 3.0 Hz. Thus, the anti-aliasing condition is fully satisfied at zero stiffness perturbation.

From Figure 6a,b, it can be observed that the in-phase mode frequency sensitivity in terms of change in resonant frequency ($∆\omega_{ip}=\;\omega_{ip}^{1g}-\;\omega_{ip}^{0g})$ of the resonator at 1 g with respect to 0 g input acceleration is 16.5 Hz. Similarly, the out-of-phase mode sensitivity in terms of change in resonant frequency for 3-DoF weakly coupled resonators is $∆\omega_{op}=\;\omega_{op}^{1g}-\;\omega_{op}^{0g}$ for 1 g input acceleration is 14.5 Hz. Thus, the relative sensitivity in parts per million (ppm) $i.e.,\;[(∆\omega_{op/ip}/\omega_{op/ip}^{0g})\times 10^{6}]\;$for 3-DoF weakly coupled resonators in in-phase and out-of-phase mode is 1478.78 ppm/g and 1299.18 ppm/g respectively.

### 4.5. Amplitude Ratio of 3-DoF Resonators as Sensitivity Metric

**Figure 7:**
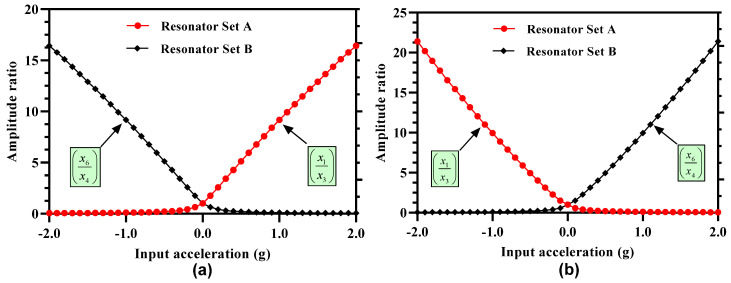
Amplitude ratio of the outer and inner resonators in 3-DoF resonators Set A and Set B (**a**) in-phase mode frequency (**b**) out-of-phase mode frequency.

The amplitude ratios of the outer and inner resonators in the resonator Set A and Set B are obtained at both the in-phase and out-of-phase mode frequency and at 35 V DC and 15 mV AC voltage for the input acceleration in the range of ±2 g. Figure 7a shows the variation in the amplitude ratios of the resonators in the two sets at in-phase mode frequency. The results show that for resonator Set A, the amplitude ratio (x1x3) is nearly zero for a negative stiffness perturbation corresponding to input acceleration in the range of −0.5 g to −2 g and increases non-linearly for small stiffness perturbation values in the acceleration range of +0.5 g to −0.5 g. However, for an input acceleration in the range of 0.5 g to 2 g, the amplitude ratio increases linearly with a maximum value of 16.43 at input acceleration of 2 g. The amplitude ratio of outer and inner resonator in Set B i.e., (x6x4) follows a symmetrical and opposite behavior with respect to stiffness perturbation in comparison to the resonator Set A. Figure 7b shows the amplitude ratios of the outer and inner resonators at out-of-phase mode. The amplitude ratio of resonators in Set A is nearly zero for positive stiffness perturbation 0.5 g to 2 g and increases linearly for the negative stiffness perturbation in the input acceleration range of 0 to 2 g. The variation of amplitude ratio of the resonators in the Set B is symmetrical with respect to the resonators in Set A. The maximum value of amplitude ratio for the resonators at out-of-phase mode is 21.4 which is higher than the corresponding amplitude ratio value at in-phase mode frequency. Thus, for the subsequent analysis for the proposed MEMS accelerometer design, we have considered the out-of-phase mode frequency as the operational frequency. The amplitude ratio of the resonators in Set A and Set B varies from 1 to 9.95 for an input acceleration in the range of 0 to ±1 g which shows an absolute amplitude ratio sensitivity (AR/g) of 8.95 for two resonator sets. The relative value of the amplitude ratio sensitivity ($\mathrm{AR}/g\times 10^{6}$) is $8.95\times 10^{6}$ ppm/g for the MEMS accelerometer design which is 6889 times higher than the relative frequency sensitivity discussed in Section 4.4.

**Figure 8:**
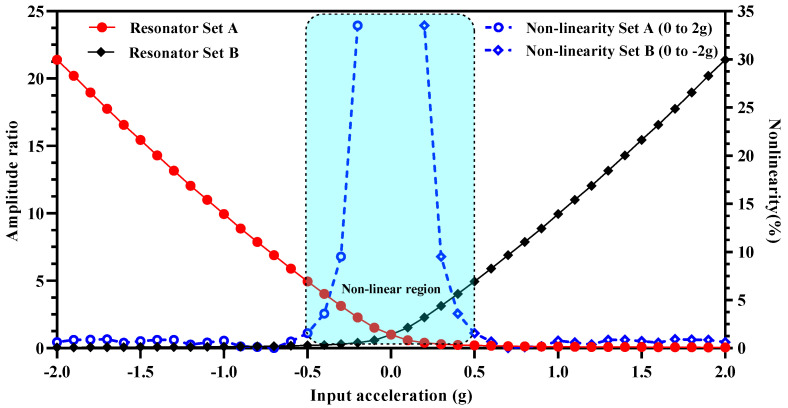
Nonlinearity in the amplitude ratio values for the 3-DoF resonators in Set A and Set B of the MEMS accelerometer.

One of the major concerns for amplitude ratio-based output metric for the weakly coupled MEMS resonators is the nonlinearity for the small values of stiffness perturbation [18,22]. Figure 8 shows the plot for the nonlinearity in the amplitude ratio values for 3-DoF weakly coupled resonators in Set A and Set B for the out-of-phase mode frequency. The results show that for input acceleration in the range of ± 0.5 g, the nonlinearity in the amplitude ratio increases exponentially for both the coupled resonators in Set A and Set B. The maximum value of nonlinearity approaches to 39% in the input acceleration range of ±0.2 g. This nonlinearity in the amplitude ratio for small values of the input acceleration limits the measurement range of the mode-localization based resonant MEMS accelerometers.

### 4.6. Amplitude Ratio Difference of 3-DoF Resonators as Sensitivity Metric

In addition to the limitation of nonlinear response in the small input acceleration range, the amplitude ratio of the resonators in both Set A and Set B is also strongly dependent on the input acceleration being in the positive or negative range. From Figure 4 and Figure 7, it is clear that for the 3-DoF resonator Set A and for input acceleration in the range of 0 to −2 g (∆k/k < 0) the out-of-phase mode amplitude ratio should be selected for high sensitivity and for input acceleration in the range of 0 to 2 g (∆k/k > 0) the amplitude ratio of the in-phase mode should be selected. Similarly, for the resonators Set B, the operational mode for amplitude ratio must be switched between the in-phase and out-of-phase mode for high sensitivity depending on the normalized stiffness perturbation being negative or positive. The selection of difference of amplitude ratios of the 3-DoF weakly coupled resonators sets allows to mitigate the limitations of using the amplitude ratios of individual resonator Set A and Set B as an output metric.

**Figure 9:**
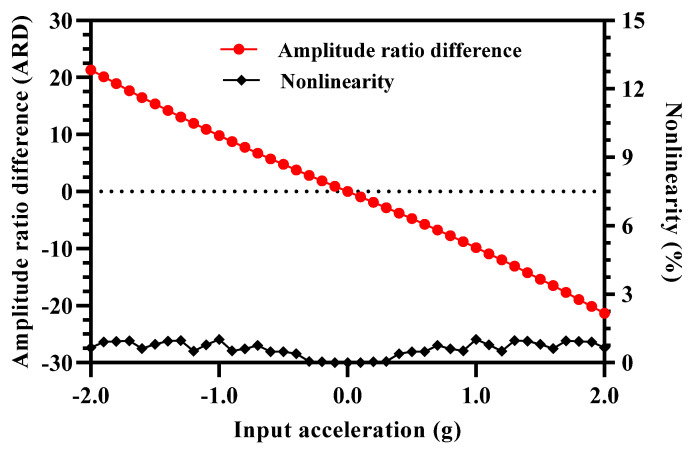
Amplitude ratio difference of resonators in Set A and Set B of MEMS accelerometer and nonlinearity for input acceleration in the range of ±2 g.

Figure 9 shows the amplitude ratio difference graph for the MEMS accelerometer design in the input acceleration range of ±2 g. The results show that the difference of the amplitude ratio of 3-DoF resonators in Set A and Set B varies linearly with an increase in the input acceleration. The nonlinearity values are less than 0.5% for the input acceleration range of ±2 g. This shows that for the MEMS accelerometer design, the amplitude ratio difference as sensitivity metric overcomes the limitation of the nonlinearity in the input acceleration range of 0 to ±0.5 which is present when only amplitude ratio of resonators in Set A and Set B is considered. In the input acceleration range of 0 to ±1 g, the amplitude ratio difference varies from 0 to 9.84 which shows an absolute amplitude ratio difference sensitivity (ARD/g) of 9.84.

## 5. Sensitivity Tuning of MEMS Accelerometer

From Equations (18), (21) and (22) it is clear that both the amplitude and frequency response of the proposed MEMS accelerometer design are strongly dependent on the mechanical beam stiffness of inner/outer resonators, middle resonators and mechanical coupling springs. In the present design, the value of mechanical coupling spring is fixed while electrostatic tuning plates are attached to the 3-DoF resonators in both the Set A and Set B. These tuning electrodes allow to tune the sensitivity of the proposed MEMS accelerometer in a specific range.

### 5.1. Electrostatic Stiffness Tuning of Inner/Outer Resonators

As shown in Figure 1, in the MEMS accelerometer design, electrostatic tuning electrodes are attached to the mechanical suspension beams of inner and outer resonators of the 3-DoF resonator Set A and Set B. For the static and dynamic response results, discussed in Section 4, the net voltage applied to these tuning electrodes is zero and thus only mechanical stiffness of beams is present. However, by applying the tuning voltage to these electrodes, a negative stiffness change in the inner and outer resonators occurs due to electrostatic spring softening effect and can be estimated as;

(29) $$∆k_{T}=-\frac{N_{te}\varepsilon A_{te}}{{\left(d_{TE}\right)}^{3}}{\left(∆V\right)}^{2}$$

**Figure 10:**
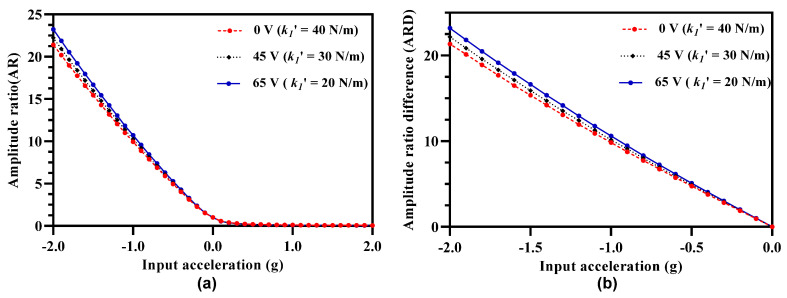
Effect of electrostatic stiffness tuning of the outer and inner resonators in both resonators sets on (**a**) amplitude ratio sensitivity (**b**) amplitude ratio difference sensitivity.

As discussed previously in [20] and also evident from Equations (14) and (23), both the amplitude and frequency response of the 3-DoF weakly coupled resonators is strongly dependent on the effective stiffness of the resonators. Figure 10a shows the effect of increasing tuning voltage on the amplitude ratio of outer and inner resonators in resonator Set A. It can be observed that with the increase in the tuning voltage, the amplitude ratio values increase for the input acceleration in the range of ±2 g. Figure 10b shows the same effect for the amplitude ratio difference of the resonator Set A and Set B with an increase in the tuning voltage. It should be noted here that for the 3-DoF resonator sets, the maximum value of tuning voltage that can be applied to the outer and inner resonators is 65 V since increasing the voltage beyond this value violates the condition given by Equation (25).

**Figure 11:**
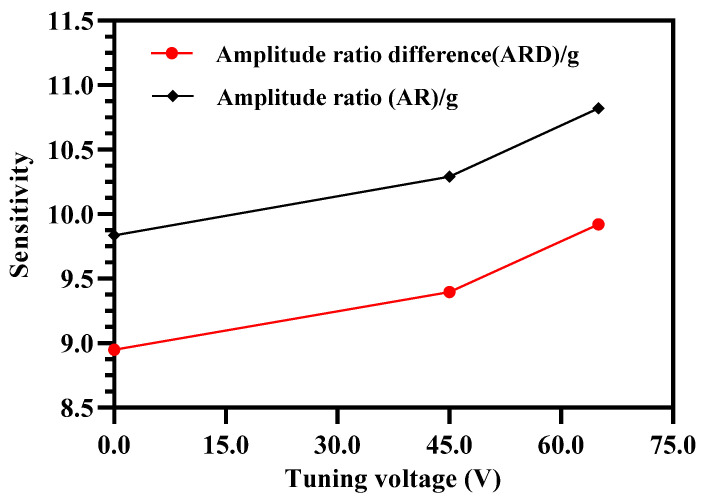
Effect of applying electrostatic tuning voltage on the outer and inner resonators (in Set A and Set B) on the amplitude ratio and amplitude ratio difference sensitivity.

Figure 11 summarizes the effect of increasing tuning voltage on the amplitude ratio and amplitude ratio difference sensitivity of the MEMS accelerometers. The amplitude ratio and amplitude ratio difference sensitivity increases to 9.92 and 10.82 respectively at tuning voltage of 65 V in comparison to the amplitude ratio and amplitude ratio difference sensitivity values of 8.94 and 9.84 respectively at 0 V.

**Figure 12:**
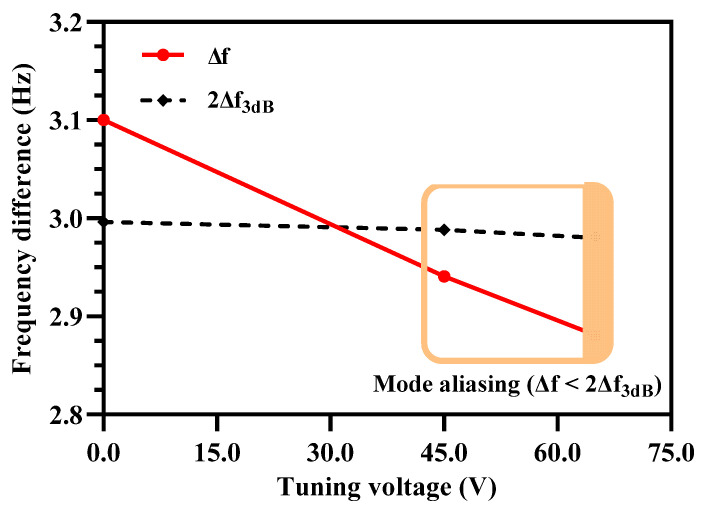
Effect of increasing tuning voltage on outer/inner resonators in Set A and Set B on the mode aliasing.

As discussed in Section 4.4, to avoid mode aliasing effect the condition of ∆f > $2∆f_{3dB}$ must be satisfied. The electrostatic stiffness tuning of the outer and inner resonators in Set A and Set B of the resonators though results in an increase in the sensitivity, it may result in the violation of the mode aliasing condition. The effect of applying electrostatic tuning voltage to the coupled resonators on shift in out-of-phase resonant frequency is analyzed for both the resonator Set A and Set B through FEM simulations. Figure 12 shows that for the resonator Set A, the increase in the tuning voltage and corresponding decrease in the effective stiffness of the outer/inner resonators violates the basic condition of ∆f > $2∆f_{3dB}$. At tuning voltage of 45 V, the ∆f = 2.941 Hz and $2∆f_{3dB}$ at out-of phase mode is 2.988 Hz. Similarly at tuning voltage of 65 V, the ∆f = 2.88 Hz and $2∆f_{3dB}$ at out-of phase mode is 2.948 Hz. 

### 5.2. Electrostatic Stiffness Tuning of Middle Resonators in 3-DoF Weakly Coupled Resonators

**Figure 13:**
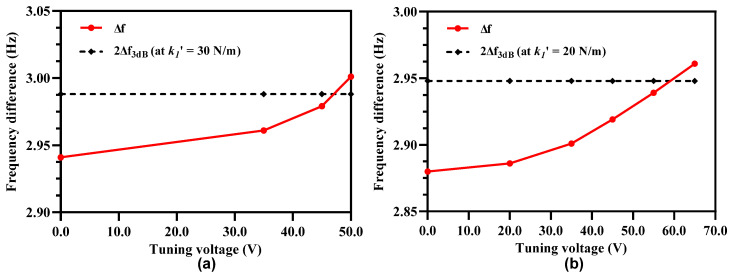
Effect of applying electrostatic tuning voltage to the middle resonators in 3-Dof resonator Set A and Set B on the frequency separation between two modes for (**a**) $k_{1}^{\prime }$ = 30 N/m and (**b**) $k_{1}^{\prime }$ = 20 N/m.

The frequency separation between the in-phase and out-of-phase resonant modes of the 3-DoF weakly coupled resonator sets in the MEMS accelerometer and hence the mode aliasing effect is dependent on the mechanical stiffness of the resonators and coupling springs with a relation represented by the γ parameter (Equation (23)) in Equations (21) and (22). The electrostatic mechanical stiffness tuning of the outer and inner resonators in the Set A and Set B of the MEMS accelerometer results in an increase in the amplitude ratio of the resonators at tuning voltage of 45 V and 65 V, but decreases the spacing between the two resonant modes which leads to mode aliasing. In this section, the effect of electrostatic stiffness tuning of the middle resonators, in both the resonators Set A and Set B, is analyzed through FEM simulations. Figure 13a shows that for the case when tuning voltage of 45 V is applied to the outer and inner resonators with $k_{1}^{\prime }$ = 30 N/m, the increase in the electrostatic tuning voltage on the middle resonators results in an increase in the ∆*f* between the in-phase and out-of-phase resonant modes. At tuning voltage of 50 V, applied to the electrostatic tuning electrodes attached to the middle resonators, the ∆*f* = 3 Hz which is above the $2∆f_{3dB}$ value of 2.980 Hz. Similarly, Figure 13b shows that at the tuning voltage of 65 V to the middle resonators, the ∆*f* value between the two modes is 2.961 Hz which is above the $2∆f_{3dB}$ value 2.948 Hz for the case when $k_{1}^{\prime }$ = 20 N/m. These results show that by the electrostatic stiffness tuning of the middle resonators, the anti-aliasing condition for $k_{1}^{\prime }$ = 30 N/m and $k_{1}^{\prime }$ = 20 N/m can be satisfied.

**Figure 14:**
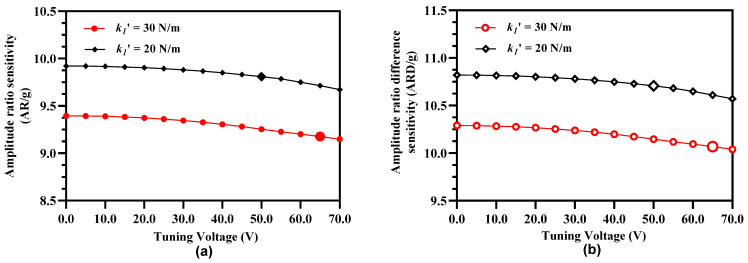
Effect of applying electrostatic tuning voltage to the middle resonators in 3-Dof resonator Set A and Set B with $k_{1}^{\prime }$ = 30 N/m and $k_{1}^{\prime }$ = 20 N/m on (**a**) amplitude ratio sensitivity and (**b**) amplitude ratio difference sensitivity.

From Equation (18), it is clear that the vibration amplitude of the outer and inner resonators in the 3-DoF weakly coupled resonator sets for a stiffness perturbation is also dependent on the mechanical stiffness of the middle resonators in Set A and Set B of the MEMS accelerometer. Thus, effect of electrostatic stiffness tuning of the middle resonator on the amplitude ratio sensitivity (AR/g) and amplitude ratio difference sensitivity (ARD/g) of the outer and inner resonators in both sets is analyzed for tuning voltage of 45 V and 65 V applied to the outer and inner resonators. Figure 14 show that for electrostatically tuned effective stiffness $k_{1}^{\prime }$ = 30 N/m and $k_{1}^{\prime }$ = 20 N/m of outer and inner resonators, the amplitude ratio and amplitude ratio difference sensitivity decreases by increasing the electrostatic tuning voltage to the middle resonator. For the case of $k_{1}^{\prime }$ = 30 N/m, the AR/g and ARD/g values are 9.255 and 10.150 respectively for a tuning voltage values of 50 V. Similarly, for the case of $k_{1}^{\prime }$ = 20 N/m, the AR/g and ARD/g values are 9.713 and 10.611 respectively for a tuning voltage value of 65 V.

### 5.3. Dynamic Range and Resolution of MEMS Accelerometer

The lower absolute value of input acceleration for the proposed MEMS accelerometer design is limited by the nonlinearity and mode-aliasing effect. As discussed in Section 4.5, for input acceleration ≤±0.5 g, the amplitude ratio of outer and inner resonators in both resonator Set A and Set B is highly nonlinear. Thus, limiting the dynamic input acceleration range to ±0.5 g to ±2 g if amplitude ratio is considered as an output metric for acceleration measurement. However, when amplitude ratio difference is considered as an output metric, the overall response becomes linear for the whole input acceleration range of ±2 g. Thus, the proposed MEMS accelerometer design allows to overcome the inherent nonlinearity effect on the dynamic input acceleration range by considering the amplitude ratio difference of two resonator sets as an output metric instead of amplitude ratio of outer and middle resonators in individual resonator sets.

As discussed in Section 5.1, by using electrostatic stiffness tuning the sensitivity of the MEMS accelerometer increases but the both in-phase and out-of-phase modes become aliased. This, effectively limits the lower value of measurable input acceleration to ± 0.05 g for $k_{1}^{\prime }$ = 30 N/m and $k_{1}^{\prime }$ = 20 N/m. This limitation of mode aliasing effect of lower value of measurable input acceleration is overcome by using electrostatic stiffness tuning of the middle resonators as shown Figure 13. Thus, the mode-aliasing is effectively removed for the 3-DoF weakly coupled resonators in both Set A and B of the MEMS accelerometer in the input acceleration range of ±0.05 g.

**Figure 15:**
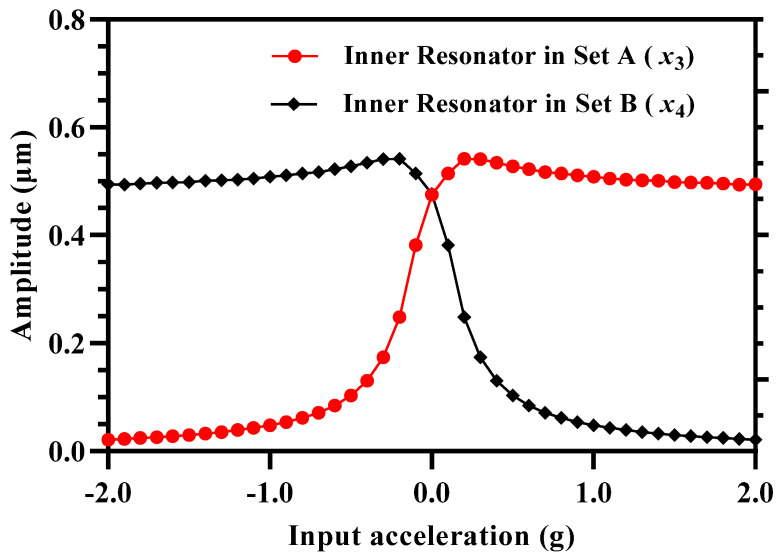
Vibration amplitude of inner resonators in both 3-DoF resonator Set A and Set B with increasing value of input acceleration.

The upper value of the dynamic input acceleration is limited by the minimum amplitude the resonator that can be measured. For the 3-DoF weakly coupled resonators, the increase in the input acceleration and hence perturbation in the inner resonators, that are electrostatically coupled to the proof mass in both Set A and Set B, leads to a decrease in the vibration amplitude as shown in Figure 15. For an input acceleration in the range of 0 to −2 g, the perturbation in the inner resonator in Set A results in a decrease in the vibration amplitude $x_{3}$. Similarly, an input acceleration in the range of 0 to +2 g and corresponding stiffness perturbation in the inner resonator in Set B results in a decrease in the vibration amplitude $x_{4}$.

The minimum detectable amplitude for a resonator is limited by the amplitude fluctuations due to noise in the system. The thermomechanical noise developed due to dynamic equilibrium between the mechanical energy of the resonator and thermal energy of the surroundings is considered to be the fundamental noise factor for the MEMS resonators within 3 dB bandwidth [20]. Assuming that the noise in the inner resonators in both Set A and Set B is Gaussian and 3 dB bandwidth for a resonator is very less than the resonant frequency $i.e.,\;∆f_{3dB}<<f_{r}$, displacement in the resonator caused only by the thermomechanical noise can be expressed as [23]

(30) $$x_{r}^{noise}=\sqrt{\frac{4k_{b}T∆f_{3dB}Q}{m_{r}\omega_{r}^{3}}}$$

where $k_{b}$ is Boltzmann constant, T is ambient temperature, Q is quality factor, $m_{r}$ is effective modal mass and $\omega_{r}$ is the angular resonant frequency of resonator. From Equation (30), the noise equivalent displacement obtained for the inner resonators in Set A and Set B of the MEMS accelerometer is $1.22\times 10^{-5}$ μm. The minimum amplitude of the inner resonators in Set A ($x_{3})$ and Set B ($x_{4})$ is 0.02 μm at 2 g input acceleration. This shows that signal-to-noise ratio at lowest amplitude of the resonators in the MEMS accelerometer design very high and is equal to 1643. 

The resolution of the MEMS accelerometer design can be calculated by estimating the minimum resolvable shift in the inner resonators stiffness due acceleration induced perturbation by using following expression [24].

(31) $${\left(\frac{∆k}{k}\right)}_{min}=8\frac{k_{c}}{k}\sqrt{\frac{k_{b}T∆f_{3dB}}{2m_{r}\omega_{r}^{3}x_{r}{}^{2}Q}}$$

For the case for $k_{1}^{\prime }$ = 20 N/m and tuning voltage of 65 V applied to the middle resonators, the minimum resolvable shift in the stiffness for the inner resonators in both Set A and Set B is $6.06\times 10^{-10}$. From this value, the resolution of the proposed MEMS accelerometer design in terms of input acceleration can be calculated as;

(32) $$\mathrm{Resolution}=\frac{{\left(∆k/k\right)}_{min}}{\left(∆k/k\right)/g}=0.22\;\mu g/\sqrt{\mathrm{Hz}}$$

## 6. Discussion

### 6.1. Comparison of 3-DoF Weakly Coupled MEMS Resonators Based Accelerometer Designs

Table 2 shows the comparison of the MEMS accelerometer presented in this work with the other accelerometers designs presented in the literature using mode localization concept. Most of the designs presented in the literature convert the input acceleration range of ±1 g with amplitude ratio as output metric for the acceleration measurement. For applications like structural health monitoring [25], hand tremor detection [26,27], and earthquake sensing [28], the MEMS accelerometer should be able to detect input acceleration up to ±2 g. One other parameter which is generally not presented in the mode-localization based MEMS accelerometers presented in the literature is overall size of the MEMS accelerometer. Most of the designs listed in Table 2 utilize two mass system for acceleration measurement except in [18] which utilizes single proof mass. The two mass system results in an increase in the overall size of the MEMS accelerometer. In comparison to the mode-localization based MEMS accelerometers, the 3-DoF weakly coupled MEMS resonators-based accelerometer design presented in this work offers large input dynamic range, high linearity, high sensitivity, high resolution with comparatively small size. 

### 6.2. Comparison of 3-DoF and 2-DoF Weakly Coupled MEMS Resonators Based MEMS Accelerometer Designs

**Figure 16:**
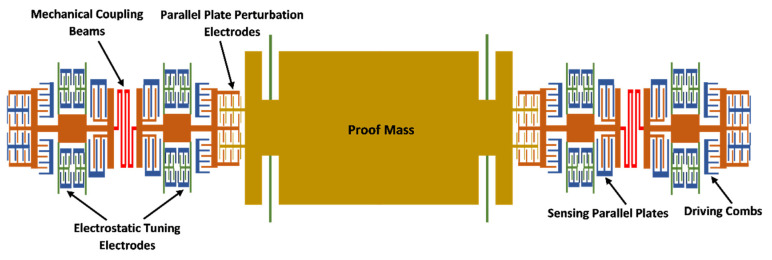
The structural design of mode-localized MEMS accelerometer with two sets of 2-DoF weakly coupled resonators on either side of central proof mass.

**Figure 17:**
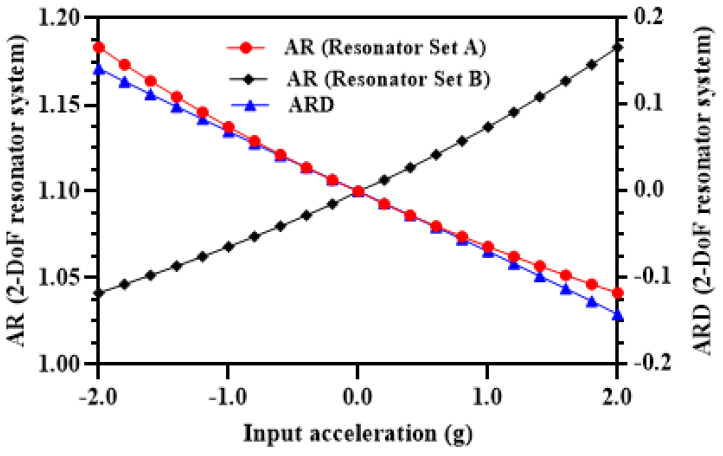
The amplitude ratio and amplitude ratio difference of 2-DoF weakly coupled resonators based MEMS accelerometer.

In this section, the comparison of the amplitude ratio and amplitude ratio sensitivity of the 3-DoF resonator based MEMS accelerometer is presented with respect to the 2-DoF weakly coupled resonator system, shown in Figure 16, at the same testing conditions discussed in Section 4.2. Figure 17 shows the amplitude ratio and amplitude ratio difference for 2-DoF weakly coupled resonators based MEMS accelerometer for an input acceleration range of ±2 g. The results show that the amplitude ratio sensitivity for the 2-DoF resonator system based MEMS accelerometer is only 0.137/g which is 65 times less than the AR sensitivity value for the un-tuned 3-DoF resonator system based accelerometer. The ARD sensitivity value for the 2-DoF resonator system based design is 0.069/g which is 128 times less than the 3-DoF resonator system based MEMS accelerometer. The mechanical structure of the proposed 3-DoF weakly coupled resonators based MEMS accelerometer is relatively complex involving multiple masses and mechanical suspensions. However, the proposed 3-DoF system allows to achieve higher sensitivity in comparison to the conventional 2-DoF weakly coupled resonator systems.

### 6.3. Effect of Microfabrication Process Tolerances on the MEMS Accelerometer

**Figure 18:**
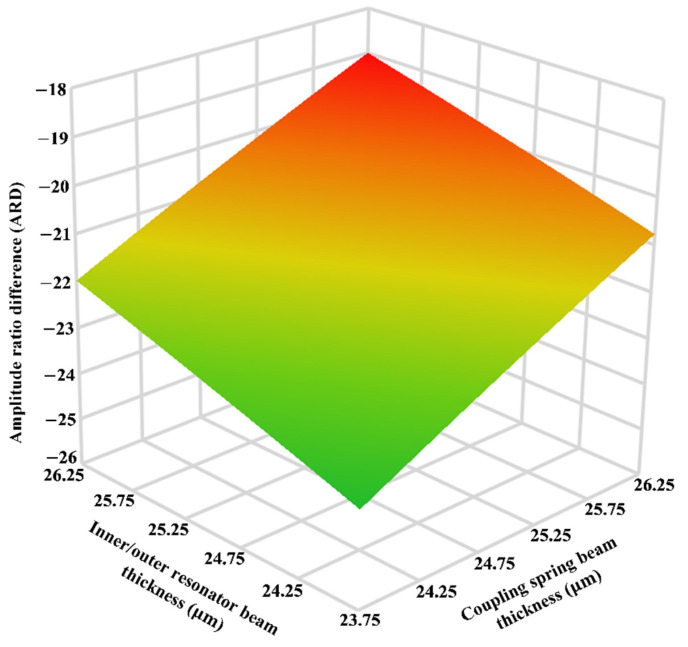
The response surface plot for the effect of the ±5% variation in the beams thickness of inner/outer resonators and coupling springs on ARD of the MEMS accelerometer.

Since, the proposed MEMS accelerometer design involves multiple masses that are coupled together through mechanical suspension beams, any process variation can drastically effect the device performance parameters. The SOIMUMPs microfabrication process is a relatively matured foundry process with process tolerances of only ±1 µm. In this section, the effect of these process tolerances on the variation in the stiffness of the mechanical suspension beams of the three weakly coupled resonators and hence on the performance parameters of the proposed 3-DoF weakly coupled resonators based MEMS accelerometer is analyzed. Table 3 shows the values of the spring constant of the inner/outer ($k_{1}$), middle resonators ($k_{2}$) and coupling spring ($k_{c}$) at nominal thickness of 25 µm and with ±5% variation in thickness due to SOIMUMPs process tolerances. The effect of variations in the spring beams thickness on the main performance parameters including AR, ARD, ∆f and $2∆f_{3dB}$ is analyzed through design of experiments (DOE) based response surface methodology (RSM) [29,30]. The spring constant of three resonators are considered as design factors with each being at three levels. Table 4 show the central composite design (CCD) based simulation matrix with 16 different combinations of thickness values for resonators and coupling spring beams and corresponding output responses at input acceleration of 2 g. A regression analysis is carried out for the output responses which showed that the interaction between the $k_{1}$ and $k_{c}$ is most significant. Figure 18 shows the response surface plot for the effect of the ±5% variation in the beams thickness of inner/outer resonators and coupling springs on ARD of the MEMS accelerometer. The value of middle resonator thickness is considered to be at nominal value of 25 µm The results show the ARD values change linearly with the variation in the inner/outer resonators and coupling spring beam thickness from 23.75 µm to 26.25 µm. At inner/outer resonators and coupling spring beam thickness of 23.75 µm and 26.25 µm, ARD values are 23.94 and 18.99 respectively. This shows that the variation in the ARD with respect to nominal thickness of 25 µm (ARD = 21.34 at 2 g) for mechanical beams is 10.9%.

**Figure 19:**
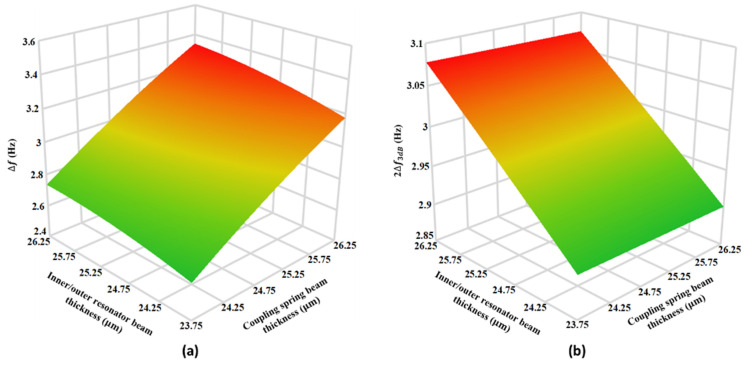
The response surface plots for the effect of the ±5% variation in the beams thickness of inner/outer resonators and coupling springs on (**a**) ∆f and (**b**) $2∆f_{3dB}$.

Figure 19a shows the effect of 5% variation in the mechanical suspension beams of the inner/outer resonators and coupling springs on the frequency difference between the in-phase and out-of-phase mode at nominal thickness value of 25 µm for the middle resonator beams. The results show that doe low thickness value of 23.75 µm for inner/outer resonators and coupling springs and hence low stiffness values, the ∆f is at minimum value of 2.501 Hz. However, with an increase in the resonators and coupling spring thickness to 26.75 µm, the frequency separation increase to maximum value of 3.502 Hz. The change in the ∆f value due to ±5% variation in beam thickness value of the resonators and coupling springs with respect to nominal thickness is nearly 14.3%.

Figure 19b shows the effect of 5% variation in the inner/outer resonators and coupling springs beam thickness on the $2∆f_{3dB}$. At the low values of beam thickness for both resonators and coupling spring i.e., at 23.75 µm, the $2∆f_{3db}$ value is 2.976 Hz by considering the ∆f value of 2.501 Hz at beam thickness of 23.75 µm from Figure 19a, it can be observed that mode aliasing condition is violated. Moreover, Figure 19b shows that at the resonators and coupling spring thickness of 26.25 µm, the $2∆f_{3dB}\;$= 2.944 which is less than corresponding ∆f value of 3.502 Hz shown in Figure 19a. the results show that due to process variations based −5% thickness change with respect to nominal thickness value of 25 µm will lead to high AR and ARD sensitivity but result in severe mode aliasing. As discussed in earlier in Section 5.2, this mode aliasing can be eliminated by the electrostatic tuning of the middle resonator stiffness. In this present case, with −5% variation in the resonators and coupling spring beam thickness, an electrostatic tuning voltage of 100 V to the middle resonator results in new ∆f value of 3.0 Hz which is higher than corresponding $2∆f_{3dB}$ value of 2.976 Hz. Thus, mode aliasing condition is satisfied.

### 6.4. Effect of Operating Temperature on Performance of the MEMS Accelerometer

**Figure 20:**
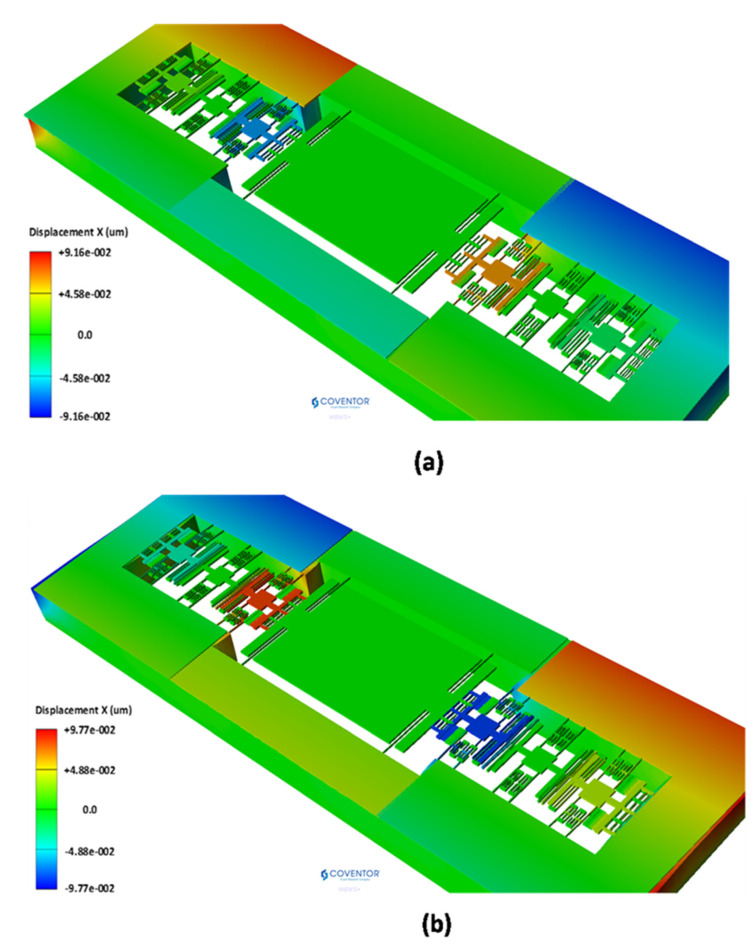
Thermal mechanical deformation profile of the MEMS accelerometer structure at (**a**) −40 °C and (**b**) 85 °C.

The operating temperature of the MEMS accelerometer is generally in the range of −40 °C to 85 °C [31]. The temperature dependent properties of the material and thermal stresses affect the performance parameters of MEMS accelerometer. The temperature dependent variation in the material properties specially the Young’s modulus is very less with a 0.016% shift in value with respect to room temperature in the temperature range of −40 °C to 85 °C [32,33]. Thus, the effect of temperature dependent variation in Young’s modulus is ignored in the subsequent discussion. To analyze the effect of operating temperature variation, in the range of −40 °C to 85 °C, on the performance of 3-DoF weekly coupled MEMS accelerometer, an FEM based thermomechanical analysis is carried out. Both the silicon substrate and structural layer of 400 µm and 25 µm thickness are considered in the analysis. The thermal deformation analysis results show that temperature induced deformation in MEMS accelerometer structure is mostly in-plane. Figure 20a and (b) shows the thermal deformation profile of the MEMS accelerometer at −40 °C and 85 °C respectively. The results show that at −40 °C, the inner resonators in both set A and set B have equal thermal deformation in opposite directions with a value of 59 nm. Similarly, the thermal deformation in the outer resonators in both set A and set B is equal and in opposite direction with a value of 17.42 nm. Figure 20b shows that at 85 °C, the inner resonator sets move towards the proof mass with a value of 83 nm, thus decreasing the initial gap between the perturbation electrodes.

**Figure 21:**
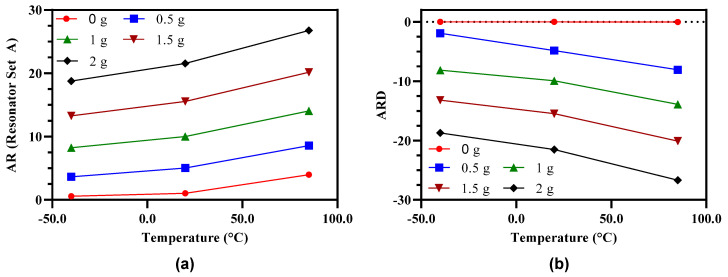
Effect of operating temperature variations on the (**a**) Amplitude ratio and (**b**) amplitude ratio difference of the MEMS accelerometer.

Figure 21a shows the effect of operating temperature variations on the amplitude ratio of the 3-DoF weakly coupled resonators in Set A of the MEMS accelerometer for input acceleration in the range of 0 to 2 g. At 0 g input acceleration the amplitude ratio of outer and inner resonators (x1x3) in Set A is 0.57 at −40 °C and 3.98 at 85 °C. This shows that due to operating temperature variations, the amplitude ratio equilibrium point is shifted due to thermal deformation of the inner/outer resonators and corresponding change in the initial gap between the capacitive perturbation electrodes. The percentage change in the amplitude ratio sensitivity with respect to room temperature at −40 °C is 14.8% and at 85 °C is 10.8%. Moreover, the difference of amplitude ratio with respect to room temperature increases nonlinearly with the increasing value of input acceleration in the range of 0 g to 2 g. Figure 21b shows the at 0 g input acceleration, the amplitude ratio difference of resonator Set A and Set B is nearly zero. This is primarily due to symmetric thermal deformation in both the resonator Set A and Set B at a given operating temperature at 0 g. However, with an increase in the input acceleration the amplitude ratio difference between the two resonator sets increases. The percentage change in the amplitude ratio difference sensitivity with respect to room temperature at −40 °C is 18% and at 85 °C is 28%. This high value of deviation for amplitude ratio difference sensitivity with respect to room temperature can be attributed to further decrease in the amplitude ratio in the resonator Set B with an application of input acceleration. The thermal deformation results for the 3-DoF weakly coupled resonators based MEMS accelerometer show that the mechanical design can be further improved to minimize the thermal deformations in the operating temperature range of −40 °C to 85 °C to achieve robustness.

## 7. Conclusions

In this paper, a new design of the MEMS accelerometer based on mode-localization in 3-DoF weakly coupled MEMS resonators is presented with enhanced input dynamic range of ±2 g and sensitivity, by following the fabrication constraints of commercially available SOIMUMPs process. The central proof mass of the MEMS accelerometer is electrostatically coupled to the two sets of 3-DoF coupled MEMS resonators. The electrostatic stiffness perturbation in the resonators coupled to the central proof mass varies linearly with an input acceleration in the range of ±2 g. Initially, the effect of input acceleration on the mode-aliasing between the in-phase and out-of-phase mode is analyzed which showed that with the nominal values of design parameters there is no mode-aliasing at 0 g. The relative frequency sensitivity values for the 3-DoF weakly coupled resonators in in-phase and out-of-phase mode are obtained as 1478.78 ppm/g and 1299.18 ppm/g respectively. The amplitude ratio of the outer and inner resonators in both the 3-DoF resonator sets is obtained as 1 at 0 g with maximum amplitude of 0.44 μm for both resonators. The results showed that with an increase in the input acceleration, the amplitude ratio between the outer and inner resonators in a resonator set increases. The amplitude ratio of resonators in out-of-phase mode is higher with a maximum value of 21.4 in comparison to in-phase mode in which maximum value is 16.4 at the maximum input acceleration of ±2 g. Thus, for the MEMS accelerometer design, out-of-phase mode is chosen as a working mode for higher sensitivity. The relative value of the amplitude ratio sensitivity ($\mathrm{AR}/g\times 10^{6}$) obtained for the MEMS accelerometer design is $8.95\times 10^{6}$ppm/g which is 6889 times higher than the relative frequency sensitivity. It is observed that for low input acceleration values i.e., for ± 0.5 g, the amplitude ratio response is highly nonlinear with a maximum value of nonlinearity approaching to 39% in the input acceleration range of ± 0.2 g. By considering the difference of amplitude ratios of the two 3-DoF weakly coupled resonator sets, the output response is linear for the low value of input acceleration also. Thus, increasing the input dynamic range of the MEMS accelerometer to full range between ±2 g. The absolute amplitude ratio difference sensitivity (ARD/g) for the MEMS accelerometer is obtained as 9.84.

Both the amplitude ratio and amplitude ratio difference sensitivity of the MEMS accelerometer are enhanced by the electrostatic stiffness tuning of the outer and inner resonators of the 3-DoF resonator sets but results in mode aliasing. The anti-aliasing condition is thus satisfied by electrostatic tuning of the middle resonators. At a tuning voltage of 65 V, applied to the resonators in both the 3-DoF resonator sets, the absolute amplitude ratio and amplitude ratio difference sensitivity values for the MEMS accelerometer are obtained as 9.713 and 10.611 respectively which are higher in comparison to the initial amplitude ratio and amplitude ratio difference sensitivity values of 8.95 and 9.84 respectively. By considering the thermomechanical noise as main noise factor, the signal-to-noise ratio of 1643 is obtained for the maximum input acceleration value of ±2 g and for minimum vibration amplitude of the perturbation resonators. The resolution of the MEMS accelerometer in terms of input acceleration is obtained as $0.22\;\mu g/\sqrt{\mathrm{Hz}}$. The performance of the 3-DoF and 2-DoF weakly coupled MEMS resonators based accelerometers is compared which showed that the sensitivity of 3-DoF resonators based accelerometer is 128 times higher than 2-DoF resonators based MEMS accelerometer. The effect of microfabrication process tolerances on the sensitivity of the MEMS accelerometer showed a 14.3% variation in sensitivity for ±5% variation in the thickness of resonators and coupling springs beams. The effect of operating temperature variations on the proposed MEMS accelerometer design showed that at −40 °C and 85 °C, there is 10.8% and 28% change in amplitude ratio and amplitude ratio sensitivity respectively with respect to the room temperature. Thus, the mode-localization based MEMS accelerometer design presented in this paper can be used for high sensitivity seismic applications at room temperature but for operating temperature of −40 °C and 85 °C the thermomechanical stability should be improved to achieve stable operation.

## Figures and Tables

**Table 1:** Dimensions of the proposed MEMS accelerometer design.

Parameters	Values
Overall device area	5.58 × 1.38 mm^2^
Central proof mass dimensions	1.96 × 1.16 mm^2^
Mass of central proof mass $\left(m_{p}\right)$	1.36 × 10^−7^ Kg
Mechanical stiffness of proof mass suspension beams $\left(k_{p}\right)$	9.25 N/m
Overlap length of perturbation electrodes $(l_{p}$)	80 μm
Width of perturbation electrodes $\left(w_{p}\right)$	3 μm
Number of parallel plate perturbation electrodes (N)	8
Mechanical stiffness of inner and outer resonators $(k_{1}=k_{3}$)	40 N/m
Mechanical stiffness of middle resonator $\left(k_{2}\right)$	258 N/m
Mechanical stiffness of coupling beams $(k_{c}$)	1.06 N/m
Number of driving combs attached to inner and outer resonators	28
Number of electrostatic tuning plate pairs attached to each resonator $\left(N_{te}\right)$	16
Overlap area of electrostatic tuning plates $\left(A_{te}\right)$	1500 µm^2^
Number of parallel sensing plates attached to inner and outer resonators	2

**Table 2:** Comparison of the 3-DoF weakly coupled resonators based MEMS accelerometers performance with respect to mode-localized MEMS accelerometers presented in the literature.

Reference	DoF of Coupled Resonators	Output Metric	Dynamic Range	Sensitivity	Resolution	Size
Zhang et al. [14]	2	Amplitude ratio	±1 g	1.26/g	0.619 mg	-
Yang et al. [15]	2	Amplitude ratio	±1 g	1.32/g	7.608 $\mu g/\sqrt{\mathrm{Hz}}$	-
Kang et al. [16]	3	Amplitude ratio	±1 g	4.38/g	1.1 $\mu g/\sqrt{\mathrm{Hz}}$	-
Peng et al. [17]	4	Amplitude ratio	0-1 g	23.37/g	-	-
Pandit et al. [18]	2	Amplitude ratio difference	±1 g	6/g	-	10 × 10 mm^2^
This work	3	Amplitude ratio difference	±2 g	10.61/g	0.22 $\mu g/\sqrt{\mathrm{Hz}}$	5.58 × 1.38 mm^2^

**Table 3:** Three different levels of mechanical springs with respect to ±5% variation in beam thickness.

Thickness (µm)	23.75 ( −5%)	25 (0%)	26.25 ( +5%)
$k_{c}$	1.004 N/m	1.06 N/m	1.11 N/m
$k_{1}$	38 N/m	40 N/m	42 N/m
$k_{2}$	244.8 N/m	258 N/m	270.6 N/m

**Table 4:** The DOE based CCD design matrix for beam thickness of mechanical springs and corresponding output responses.

No.	$k_{c}$ (µm)	$k_{1}$ (µm)	$k_{2}$ (µm)	AR	ARD	∆f (Hz)	$2∆f_{3dB}\;(\mathrm{Hz})$
1	26.25	23.75	23.75	19.975	−19.909	3.401	2.956
2	26.25	23.75	26.25	21.683	−21.622	3.001	2.956
3	26.25	25	25	19.923	−19.857	3.302	2.912
4	23.75	23.75	23.75	23.105	−23.049	2.801	2.976
5	25	26.25	25	20.487	−20.423	3.102	2.956
6	25	25	25	21.396	−21.335	3.001	2.920
7	26.25	26.25	23.75	18.259	−18.187	3.502	2.944
8	26.25	26.25	26.25	19.864	−19.798	3.202	2.944
9	23.75	26.25	23.75	21.188	−21.126	2.901	2.968
10	25	25	26.25	22.259	−22.200	2.901	2.920
11	23.75	25	25	23.004	−22.947	2.701	2.932
12	25	25	23.75	20.552	−20.488	3.201	2.920
13	23.75	26.25	26.25	22.897	−22.839	2.601	2.968
14	25	23.75	25	22.399	−22.340	2.902	2.968
15	25	25	25	21.396	−21.335	3.001	2.920
16	23.75	23.75	26.25	24.858	−24.805	2.501	2.976
